# A short questionnaire to assess pediatric resident’s competencies: the validation process

**DOI:** 10.1186/1824-7288-39-41

**Published:** 2013-07-05

**Authors:** Liviana Da Dalt, Pasquale Anselmi, Silvia Bressan, Silvia Carraro, Eugenio Baraldi, Egidio Robusto, Giorgio Perilongo

**Affiliations:** 1 Paediatric Residency Program, Department of Woman’s and Child’s Health, University of Padua, Via Giustiniani, 3 – 35128 Padua, Italy; 2 Department FISPPA, University of Padua, Via Venezia 8, 35131 Padua, Italy

**Keywords:** Resident, Pediatrics, Evaluation, Medical residency

## Abstract

**Background:**

In order to help assess resident performance during training, the Residency Affair Committee of the Pediatric Residency Program of the University of Padua (Italy) administered a Resident Assessment Questionnaire (ReAQ), which both residents and faculty were asked to complete. The aim of this article is to present the ReAQ and its validation.

**Methods:**

The ReAQ consists of 20 items that assess the six core competencies identified by the Accreditation Council of Graduate Medical Education (ACGME). A many-facet Rasch measurement analysis was used for validating the ReAQ.

**Results:**

Between July 2011 and June 2012, 211 evaluations were collected from residents and faculty. Two items were removed because their functioning changed with the gender of respondents. The step calibrations were ordered. The self evaluations (residents rating themselves) positively correlated with the hetero evaluations (faculty rating residents; Spearman’s ρ = 0.75, *p* < 0.001). Unfortunately, the observed agreement among faculty was smaller than expected (Exp = 47.1%; Obs = 41%), which indicates that no enough training to faculty for using the tool was provided.

**Conclusions:**

In its final form, the ReAQ provides a valid unidimensional measure of core competences in pediatric residents. It produces reliable measures, distinguishes among groups of residents according to different levels of performance, and provides a resident evaluation that holds an analogous meaning for residents and faculty.

## Background

The process of evaluation is a central aspect of the development and implementation of any training activity and, more specifically for this context, of all aspects of physician competence [1]. A quality educational curriculum provides formative assessment in order to document resident progress, or lack thereof, along the established learning pathway and achievement of learning objectives. Furthermore, if properly planned, orchestrated and implemented, the assessment process serves to provide feedback to trainees regarding their own level of professional and cultural maturation, and it provides the faculty with feedback on the strengths and weaknesses of the overall training program. Ideally, it should also serve to guide residents along different professional career paths. Finally, it serves to protect the public by identifying physicians who are not prepared to practice at an independent level.

The Paediatric Residency Program of the University of Padua is a Ministerial accredited 5-year program that provides a Diploma of Specialization in Paediatrics. It is also one of the largest training programs in Italy with an average of 90 residents. Approximately 80% of learning activities take place in the clinical setting practice under attending faculty supervision with the goal of increasing levels of responsibilities throughout training. The remaining learning activities include formal lectures, seminars, workshops and personal studies. Residents rotate through 15 of the 25 Divisions/Services of the Department of Woman’s and Child’s Health of Padua and of the affiliated Hospitals during their first three years; rotations range in time from three to six months. During the last two years of training residents select elective rotations involving at most three divisions, each lasting from six to twelve months. The first three years of the program are meant to provide a common cultural and professional background in general paediatrics, and the last two consolidate their knowledge and experiences with a progressive assumption of clinical responsibilities and shape their career into specific areas of paediatric practice (ie, primary, secondary, or sub-specialty care). The Staff of the Divisions/Services function as resident mentors during their rotations. During an academic year each resident has as many faculty members assigned to them as they do rotations.

The evaluation of medical residents is a complex process. In North America, it is largely based on a model developed by the North American Accreditation Council for Graduate Medical Education (ACGME), which uses six interrelated domains of competence: medical knowledge, patient care and procedural skills, professionalism, interpersonal and communication skills, practice-based learning and improvement, and Systems-Based Practice [2-4]. Multiple-choice questions are probably the most used tool to assess medical knowledge^a^, but are limited in their ability to measure all aspects of resident competency; therefore, different tools have been proposed to assess the other medical competences identified by the ACGME [5-10]*.* Indeed, the ideal processes to evaluate residents in a comprehensive manner should encompass the use of different tools to evaluate the various competences contributing to the profile of a “good pediatrician”. The use of multiple methods of assessment can overcome the limitation of individual assessment formats. Furthermore, this ideal process is expected to work better in those settings permeated by the culture of evaluation and where faculty and residents are fully aware of the relevance of a robust, standardized assessment process during training.

In Italy, medical schools have autonomy regarding the methods and standards of assessment in order to ensure consistency between the curriculum and the assessment. Furthermore, with the exception of the assessment of medical knowledge, ^b^ the assessment of other competencies is neglected or performed using “home-made” non-validated tools, or is based on the individual program staff’s personal judgments. However, this scenario is expected to change quite rapidly given the need to develop a robust, standard assessment program that will provide objective evidence to support the decision to advance residents along their educational pathway, thereby allowing them to acquire progressive levels of independence and clinical responsibilities.

With these issues in mind, the Residency Affair Committee (RAC) of the Paediatric Residency Program of the University of Padua decided to address the issue of assessing residents’ competence (other than medical knowledge) using a simple questionnaire - The Resident Assessment Questionnaire (ReAQ) - that can be self-administered by residents and/or completed by mentoring faculty (see Table 1 for a listing of the items on the ReAQ). The definition of competence in medicine elaborated by Epstein and Hundered was the one used by the RAC to conceive this questionnaire:

“the habitual and judicious use of communication, knowledge, technical skills, clinical reasoning, emotions, values, and reflecting in daily practice for the benefit of the individuals and communities to be served” [11].

**Table 1:** The Resident assessment questionnaire (ReAQ)

		**Poor**	**Mediocre**	**Respectable**	**Good**	**Excellent**
	PROFESSIONALISM					
1	Attention to ethnic and cultural diversities	1	2	3	4	5
2	Leadership	1	2	3	4	5
3	Confidentiality in dealing with clinical problems	1	2	3	4	5
4	Responsibilities	1	2	3	4	5
5	Determination, precision, reliability in pursuing the entrusted tasks	1	2	3	4	5
6	Curiosity, creativity, initiative*	1	2	3	4	5
	PATIENT CARE					
7	Accuracy in collecting medical histories and in performing physical examination	1	2	3	4	5
8	Ability to identify elements relevant to formulate diagnostic and therapeutic plans	1	2	3	4	5
9	Accuracy in the production and management of clinical documentation (including the discharge letter)	1	2	3	4	5
10	Autonomy in managing clinical-care problems	1	2	3	4	5
11	Ability in using information technologies to improve learning and care delivering	1	2	3	4	5
12	Autonomy level in performing procedures*	1	2	3	4	5
	INTERPERSONAL AND COMMUNICATION SKILLS					
13	Clarity in the presentation of clinical case	1	2	3	4	5
14	Ability to relate with medical team	1	2	3	4	5
15	Ability to relate with the child and the family	1	2	3	4	5
16	Attitude to the teamwork	1	2	3	4	5
	MEDICAL KNOWLEDGE					
17	Level of basic medical knowledge	1	2	3	4	5
	PRACTICE-BASED LEARNING AND IMPROVEMENT IN PATIENT CARE					
18	Ability to incorporate evaluations and feedbacks into the daily practice	1	2	3	4	5
19	Ability to recognize own limitations, and to ask for expert advice, when necessary	1	2	3	4	5
20	Overall judgment	1	2	3	4	5

Note: *Removed during the validation.

The major concern which inspired the design of the ReAQ was the need for a tool that was easy to use and didn’t consume a lot of time or other resources (considering the limited ones available in term of finance and human power) from the busy clinical staff.

The aim of the article is to present the ReAQ and the analyses used to secure evidence of its validity. For this purpose, a many-facet Rasch measurement (MFRM, [12]) analysis was used. Applications of Rasch models in the medical field are well documented in scientific literature [13-18]. Some advantages of these models are transformation of ordinal raw scores into interval measures, identification of poorly functioning items, generalizability of results across samples and items, and investigation of response behavior. The ultimate rationale of our work was to make available to staff members a reliable tool to evaluate the doctors they aim to educate, which ideally could be used as a model for other programs. The evaluation of residents is part of a more comprehensive evaluation system directed to assess also the faculty, the rotations, and the RAC. Ultimately, this complex system has the significance of establishing a culture of assessment, which the RAC of the Paediatric Residency Program of Padua had included among its main learning objectives.

## Methods

In constructing the ReAQ we had in mind the medical competencies listed by the ACGME other than medical knowledge [2]. The ReAQ consists of 20 items that are evaluated on a five point scale from 1 (“poor”) to 5 (“excellent”; see Table 1); the last item of the ReAQ requires an overall comprehensive judgment. The faculty and residents were given brief instructions about completing the questionnaire.

Despite a failed initial effort of administering the ReAQ via the internet, we reverted to a paper and pencil method. The faculty in charge of the Divisions/Services in which the residents were rotating were required to complete the questionnaires within three weeks of the conclusion of the rotation. The various evaluation forms were collectively evaluated by the RAC in order to arrive at a comprehensive evaluation for individual residents.

Results of this assessment, combined with scores from the American Board of Pediatrics International In-training Examination (a well-validated measure of medical knowledge; browse to http://www.abp-intl.org/intrainingexamination.html) served to express the final evaluation for that year. The results of this process were communicated to each resident by members of the RAC during individual meetings. At the end of each year, each resident had to complete the ReAQ. The aim was to provide a tool for comparing self-perceived cultural and professional acquisitions with the judgments provided by the faculty. In Italy, the academic year for residents goes from July to June. The data used for validating the ReAQ were collected between June 2010 and July 2011. Data of all residents were used for the validation. Residents of the first three years went through the rotation plan that was defined by the RAC, whereas residents of the last two years self-selected elective rotations. In the latter case, therefore, faculty were chosen by residents.

### Many-facet Rasch measurement

The MFRM ([12], see [19-22] for applications) is a formal model for transforming nonlinear, scale-dependent ordinal raw scores into linear, scale-free interval measures. In its basic form, the MFRM represents the probability *P*_*nijk*_ of a resident *n* being given by judge *j* a score *k* on an item *i* as an effect of the ability of resident *n* (β_*n*_), the difficulty of item *i* (δ_*i*_), the severity of judge *j* (γ_*j*_), and the impediment in giving a score *k* rather than *k*-1 (τ_*k*_):

$$\ln\frac{P_{\mathrm{nijk}}}{P_{\mathrm{nij}\left(k-1\right)}}=\beta_{n}-\delta_{i}-\gamma_{j}-\tau_{k}.$$

Residents, judges, and items are facets. When two or more judges evaluate each resident, and the judge pairs vary across residents, the dependence of the evaluation on the severity of judges is a concern. An important feature of the MFRM is that judge severity is estimated and removed from the measures. In the analyses that follow, the judges are both the faculty (who evaluate the residents) and the residents (who evaluate themselves). Facets concerning the gender of residents (ϵ) and judges (ζ), and the program year of the residents (η) are considered as well. The analyses are performed using the computer program Facets 3.66.0 [23].

The validation of the ReAQ has been conducted by taking into account aspects concerning the functioning of the items and of the response scale, and the dimensionality, reliability and construct validity of the questionnaire.

The functioning of the items is assessed using item mean square fit statistics (infit and outfit). Values greater than 1.4 [24] suggest that the item degrades the measurement system, or that it assesses a construct that is different from the principal one being measured (Rasch dimension). In addition to mean square fit statistics, principal component analysis of standardized residuals is used to examine whether a substantial secondary dimension exists in the residuals after the Rasch dimension has been estimated [25]. Contrasts in the residuals with eigenvalues greater than 3 are indicative of violations of the Rasch model assumption of unidimensionality [26].

Rating scale structure requires that increasing levels of performance displayed by a resident correspond to increasing probabilities that the resident will be scored in higher rating scale categories. The functioning of the ReAQ response scale is assessed by determining whether the step calibrations τ_*k*_ are ordered. If they are not, there is discordance between the category probabilities and the observed level of performance and, therefore, the response scale is not adequate for measurement purposes [27].

Reliability of the ReAQ is assessed by examining the spread of resident measures on the latent variable. Internal consistency of the ReAQ is assessed by means of the indexes separation reliability (*R*) and strata of residents. When there are not missing data, *R* is the Rasch equivalent of Cronbach’s α. Strata evaluates the number of statistically distinct groups of residents that the questionnaire is able to discern [28]. If at least two groups cannot be identified, then the questionnaire does not allow the best residents to be discerned from the worst ones. Inter-rater reliability is assessed by comparing the observed percentage of agreement among judges with that expected when their different degrees of severity are taken into account.

Validity of the ReAQ is assessed on the basis of the theoretical work of Messick [29] and Smith [30]. Messick described validity as a unitary concept, in which the traditional categories of content, criterion, and construct validity are integrated into a broad unified view of construct validity. Smith articulated how methods available in Rasch measurement can be used to address aspects of the construct validity described by Messick. Content representativeness is assessed by examining the spread of the item difficulties along the latent variable. In particular, the item strata identify the number of statistically distinct groups of item difficulties that the judges can discern. If at least two groups are unable to be identified, then the questionnaire does not allow discernment among different measurement levels of the construct. Construct generalizability is assessed using the following two methods. First, the correlations between the item measures derived from the self evaluations and those derived from the hetero (faculty) evaluations is considered in order to investigate whether the latent variable holds the same meaning for residents and faculty. Second, bias interaction analyses are performed in order to investigate whether the functioning of the items differs with the gender of judges.

The project has been approved by the Institutional ethics committee (Institution Review Board of the University Hospital of Padua).

## Results

From July 2011 and June 2012, sixty-five residents (54 F; *N* = 14, 14, 17, 18, 2 for 1st to 5th year residents, respectively) received, on the whole, 211 evaluations. Fifty-two of these were self evaluations, whereas the remaining 159 were expressed by 24 faculty (10 F). Each resident received from 1 to 6 evaluations, and each faculty evaluated from 1 to 14 residents. Given the longer duration of rotations, a smaller number of evaluations is available for the residents of the last two years. The data matrix had dimensions 211 (evaluations) × 20 (items). The MFRM analysis produced a measure for each element of each facet. Greater measures mean more positive evaluations for residents, greater difficulty (ie, fewer positive evaluations) for items, and greater severity for judges. It is worth recalling that judges are both the faculty evaluating the residents and the residents evaluating themselves.

Item 12 (“Autonomy level in performing procedures”, see Table 1) had fit statistics greater than 1.4 (infit = 1.63, outfit = 1.68). In addition, the functioning of this item and that of Item 6 (“curiosity, creativity, initiative”) changed with the gender of faculty. For both items, male judges provide more positive evaluations than female judges (*t*(144) = 3.22, *p* < 0.01 for Item 12; *t*(152) = 3.55, *p* < 0.001 for Item 6). The two items were removed, and a new analysis was run. The remaining 18 items defined a substantively unidimensional scale (the first contrast in the residuals has an eigenvalue of 2.7). The step calibrations are ordered (*t*_*poor* − *mediocre*_ = − 2.09; *t*_*mediocre* − *respectable*_ = − 1.55; *t*_*respectable* − *good*_=.13; *t*_*good* − *excellent*_ = 3.51). Therefore, the response scale has been adequately used by judges.

Table 2 contains summary statistics for the 18 items. In evaluating the residents with the ReAQ, the judges distinguished more than six groups of item difficulty (Strata = 6.64). The item measures derived from the self evaluations positively correlate with those derived from the hetero evaluations (Spearman’s ρ = 0.75, *p* < 0.001). This suggests that the ReAQ items hold the same meaning for residents and faculty.

**Table 2:** Average scores, item measures, standard errors and fit statistics of the residents assessment scale (measure order)

**Item**	**Average score**	**Measure**	***SE***	**Infit**	**Outfit**
2 [Leadership]	3.70	1.47	0.11	1.23	1.28
9 [Clinical-care problems]	3.98	0.69	0.12	1.00	0.96
3 [Confidentiality]	3.99	0.65	0.12	0.99	0.95
7 [Clinical diagnosis/therapeutic iter]	3.99	0.64	0.12	1.11	1.02
15 [Basic medical knowledge]	4.01	0.59	0.12	0.90	0.81
11 [Clinical cases presentation]	4.15	0.14	0.13	1.01	0.94
16 [Responsibility]	4.15	0.12	0.13	1.20	1.06
8 [Clinical documentation]	4.16	0.09	0.13	1.03	0.94
5 [Anamnesis/examination]	4.18	0.03	0.13	0.95	0.83
17 [Criticism acceptance]	4.19	0.00	0.13	1.10	1.05
20 [Overall judgement]	4.22	−0.14	0.13	0.94	0.84
4 [Determination]	4.25	−0.23	0.13	1.39	1.16
19 [Teamwork attitude]	4.27	−0.31	0.13	0.97	0.88
18 [Limits recognization]	4.32	−0.47	0.13	1.10	1.07
13 [Medical team relationship]	4.37	−0.67	0.14	0.96	0.92
10 [Telematic resources]	4.38	−0.71	0.14	0.87	0.92
1 [Ethnic-cultural diversity]	4.43	−0.90	0.14	1.02	0.96
14 [Child-family relationship]	4.45	−0.98	0.14	0.87	0.75

Note. Greater measures indicate more difficult items (ie, that received fewer positive evaluations).

The residents did not receive analogous evaluations (*χ*^2^(64) = 1325.3, *p* < 0.01). There were no differences between male and female residents (*e*_*male*_ = 0.05, *SE* = 0.07; *e*_*female* =_ − 0.05, *SE* = 0.03; *χ*^2^(1) = 1.3, *p* = 0.25). As expected, there were differences between residents across different program years, with residents in their last two years receiving higher evaluations. However, these differences are not reliable (*R* < 0.01), because of the limited amount of data for 5th year residents (*N* = 2).

The self evaluations were more severe than the evaluations made by faculty (average γ = 1.95, *SE* = 0.06 for residents; average γ = −0.28, *SE* = 0.11 for faculty; *z* = 18.28, *p* < 0.001). The faculty differ in severity (*χ*^2^(23) = 966.1, *p* < 0.001), and the observed agreement among them is smaller than expected (Exp = 47.1%; Obs = 41%).

The ReAQ is a reliable questionnaire, whether it is used for self evaluation (*R* = 0.90) or for hetero evaluation (*R* = 0.91). It allows for the identification of four groups of resident ability in the former condition (Strata = 4.23), and almost five groups in the latter condition (Strata = 4.69). The greater discriminative power of the hetero evaluation could be explained by considering that the faculty evaluate more than one resident, and this could help them differentiate among residents. Table 3 depicts locations of residents, judges and items on the latent variable. Greater measures for residents indicate that they received more positive evaluations, greater measures for items indicate that they were more difficult, and greater measures for judges indicate that they were more severe. Looking at the locations of residents and items on the latent variable, we can see that item difficulties are below resident evaluations. This suggest to develop new items that are more difficult (ie, items with respect to the residents receive less positive evaluations). These items are expected to further highlight differences among residents.

**Table 3:** Locations of residents, judges and items on the latent variable

**Measure**	**Resident**	**Judge**	**Item**
6.0			
5.8			
5.6			
5.4	R23		
5.2	R52		
5.0	R13 R25 R63		
4.8	R32		
4.6	R30		
4.4	R9 R10 R26 R46		
4.2	R15 R38 R39 R41 R50 R54 R59 R61 R62		
4.0	R17 R36 R37 R45		
3.8	R6 R24 R28 R29 R33 R57		
3.6	R1 R2 R7 R31 R43 R44 R53		
3.4	R12 R19 R47 R60		
3.2	R4 R21 R42 R65		
3.0	R11 R14 R20 R40 R56		
2.8	R27 R35 R58	T12	
2.6	R48		
2.4	R8 R49 R55 R64		
2.2	R5 R18	T17	
2.0	R3 R34	T13 RESIDENTS	
1.8	R16		
1.6			
1.4			[Leadership]
1.2		T14	
1.0		T5 T18	
.8	R22 R51	T20	
.6		T23 T16	[Clinical-care problems] [Confidentiality] [Clinical diagnosis/therapeutic iter] [Basic medical knowledge]
.4		T1 T19 T2	
.2			[Clinical cases presentation] [Responsibility]
.0		T4	[Clinical documentation] [Anamnesis/examination] [Criticism acceptance]
-.2		T11	[Overall judgement] [Determination]
-.4		T8	[Teamwork attitude] [Limits recognization]
-.6			[Medical team relationship]
-.8		T6 T15	[Telematic resources]
-1.0		T10	[Ethnic-cultural diversity] [Child-family relationship]
-1.2			
-1.4			
-1.6			
-1.8		T7	
-2.0		T3	
-2.2			
-2.4		T24	
-2.6			
-2.8		T22	
-3.0			
-3.2			
-3.4		T9	
-3.6			
-3.8			
-4.0		T21	

Greater measure mean more positive evaluations for residents, greater difficulty (ie, fewer positive evaluations) for items, and greater severity for judges.

## Discussions

The article presented the validation of the ReAQ, a 20 item questionnaire designed to provide information on five of the ACGME core competencies; 18 of the items well-suited for the assessment purpose.

In its final form, the ReAQ produces a valid unidimensional measure of competence. This means that, although the instrument consists of items that assess different aspects of medical practice (i.e., interpersonal relationship and communication skills, level of autonomy), all these aspects consistently contribute to the definition of the resident competence profile.

The ReAQ provides a reliable assessment whether it is used by residents for self evaluation or by faculty for resident evaluation. It permits the classification of residents into four or five levels of performance. Therefore, the ReAQ is a valid tool for distinguishing among residents at different levels of training. The resident evaluation made by the ReAQ holds an analogous meaning for residents and faculty, ie, residents and faculty substantially agree in defining strengths and weaknesses of residents.

In our data, the agreement observed between faculty was smaller than desired. To some extent, this result was expected given that residents and faculty were presented with the questionnaire without receiving any formal training about its use. As a consequence, respondents may have used subjective interpretation of what each item was requesting. In order to increase the agreement among respondents, some training and instruction are required to help them develop a shared interpretation of the items. Moreover, new items could be developed with respect to residents receive less positive evaluations. These items could contribute to further highlight differences among residents.

Literature warned researchers against the practice of misusing ordinal raw scores as they were interval measures (eg, calculating means, standard deviations and effect sizes) [31,32], and showed erroneous conclusions that can derive from applying parametric analyses inappropriately [33]. Since Rasch models allow for the transformation of ordinal raw scores into interval measures, they have been suggested as a valuable tool in both the analysis of clinical data, and the development and evaluation of instruments [30,34,35]. However, it is worth noting that Rasch models are especially demanding of data that satisfy the requirements for constructing measures. Two alternative pathways can be pursued when a Rasch model does not account for the data [35-37]. The first one consists of modifying the instrument, the definition of the variable under investigation, or both, in order to generate new data that better conform to the model. In this direction, the two items of the ReAQ whose functioning changed with the gender of faculty (Item 6 and Item 12) could be revised or replaced. The second one consists of identifying an alternative model (usually within the IRT framework) that accounts better for the given data.

The ultimate goal of this effort was to develop an effective, reliable, and widely-usable tool to conduct a comprehensive assessment of residents’ medical competence, which can help residents progress in their training pathway, and also help staff provide targeted guidance for residents. There is a large need for such assessment tools in Italy. We are fully aware that this questionnaire is not the final and comprehensive answer to the issues of residents’ evaluation and that “the various domain of medical competence should be assessed in an integrated coherent and longitudinal fashion with the use of multiple methods and provision of frequent and constructive feedbacks” [1,11,38,39]. However, it is a first step towards developing a robust and standardized program for resident evaluation. Further, the introduction of this valid tool may contribute to the development of culture of modern and effective evaluation as well as much needed research to provide a solid foundation for assessing medical education outcomes. Indeed, the validation process of the ReAQ is a pre-requisite to evaluating other components of the articulated evaluation system that the RAC of the Paediatric Residency Program of Padua has decided to implement. Future work will be devoted to investigate the association among evaluations resulting from the ReAQ, resident performances at the bedside, and patient outcomes.

## Conclusions

The ReAQ is a valid tool for resident evaluation considering that it produces reliable measures, allows the distinction of residents into different levels of performance, and holds an analogous meaning for residents and faculty. However, some training on how to use the instrument is required for respondents to properly interpret the meaning of the items and increase inter-rater reliability. In Italy, there is an increasing awareness of the relevance of an appropriate evaluation of residents. Data resulting from the application of valid tools, if shared among schools, could be used to produce additional benchmarking data to measure the performances of residents within training programs across the Country.

## Endnotes

^a^Multiple-choice examinations can provide large numbers of examination items that encompass many content areas, can be administered in a relatively short period of time, and the grading process is quick and easy.

^b^Recently some Italian paediatric residency programs have adopted the American Board of Pediatrics International In-training Examination to assess residents’ knowledge.

## Abbreviations

ACGME: Accreditation council of graduate medical education; MFRM: Many-facet rasch measurement; RAC: Residency affair committee; ReAQ: Resident assessment questionnaire.

## Competing interests

All the authors have no financial competing interests.

## Authors’ contributions

LDD and GP: Respectively, Vice-chairman and chairman of the Resident Affair Committee of the Paediatric Residency: Program of the Department of Woman’s and Child’s Health of the University of Padua – Personal involvement in designing the entire evaluation process of the program and, more specifically, of the ReAQ, in supervising its implementation, in providing feedbacks to the residents, in interpreting the data contained in the manuscript and in writing this manuscript. PA and ER: Respectively, Research Assistant and Full Professor of Psychology at the University of Padua – Personal involvement in the validation process of the ReAQ and in writing this report. SB, SC and EB: Members of the of the Resident Affair Committee of the Paediatric Residency of the Department of Woman’s and Child’s Health of the University of Padua – Personal involvement in supervising the implementation of the ReAQ, in providing feedbacks to the residents, in interpreting the data contained in the manuscript and in writing this manuscript. All authors read and approved the final manuscript.

## Authors’ information

Pasquale Anselmi – PhD in Personality and Social Psychology at the University of Padua, Italy. Eugenio Baraldi, Full Professor of Paediatrics, University of Padua, senior member of the Resident Affair Committee; Chair of the Paediatric Residency Program of Padua University since October 2012. Silvia Bressan, Assistant Professor of Paediatrics; members of the of the Resident Affair Committee of the Paediatric Residency Program of Padua (Italy). Silvia Carraro – Assistant Professor of Paediatrics; members of the of the Resident Affair Committee of the Paediatric Program of Padua. Liviana Da Dalt, Associated Professor of Pediatrics, University of Padua, Chair of the Paediatric Divisions, expert in post-graduate medical training; Vice-Chair and senior member of the Resident Affair Committee of the Paediatric Program of Padua. Giorgio Perilongo, Full Professor of Paediatrics, University of Padua, Director of the Department of Woman’s and Child’s Health of the University of Padua, Italy; Chair of the Paediatric Training Program from 2005 to September 2012; presently member of the Resident Affair Committee. Egidio Robusto Full Professor of Psychometrics at the University of Padua, Italy.
